# Liquid Crystalline π-Conjugated Copolymers Bearing a Pyrimidine Type Mesogenic Group

**DOI:** 10.3390/ma2010022

**Published:** 2009-01-16

**Authors:** Kohsuke Kawabata, Hiromasa Goto

**Affiliations:** Institute of Materials Science, Graduate School of Pure and Applied Sciences / University of Tsukuba, Tsukuba, Ibaraki, 305-8573, Japan

**Keywords:** Liquid crystal, conductive polymer, electron spin resonance

## Abstract

Phenylene-thiophene-based liquid crystalline π-conjugated copolymers bearing mesogenic groups as side chains were synthesized via a Stille polycondensation reaction and confirmed to exhibit a nematic liquid crystal phase at appropriate temperatures. The formation of a nematic phase, but not a smectic phase indicates cooperation of the main chain and side chain in the formation of a nematic main-chain/side-chain liquid crystal phase. The generation of polarons in the main chain as charge carriers during *in-situ* vapor doping of iodine is confirmed to increase with a doping progresses, exhibiting Dysonian paramagnetic behavior typical of conductive polymers.

## 1. Introduction

Liquid crystal (LC) polymers exhibit both liquid-like fluidity and crystal-like molecular order. Rigid and stick-shaped compounds with low molecular mass and rigid polymers with high molecular weight often display an LC phase under appropriate conditions. LC polymers have been applied as an engineering plastic providing good processability, strength, and elasticity [1,2]. It is generally known that the introduction of LC groups into the main chain allows polymers to form an LC phase as a side-chain LC polymer [3,4]. Rigid aromatic polymers with flexible alkyl side chains are also known to form an LC phase as a main-chain LC polymer [5,6,7,8]. In side-chain LC polymers, the main chain can form an ordered structure accompanying alignment of the side chains, and the anisotropy afforded by such ordering provides certain functionalities [9].

Conjugated polymers are also of considerable interest, having a range of useful properties including various optical properties [10], photoluminescence [11,12], and electrical conductivity [13,14]. Chemical doping with a suitable acceptor dopant such as iodine results in the generation of free radicals (polarons) on the main chain of the conjugated polymers. Polarons act as electrical conduction carriers in π-conjugated polymers, and the generation of polarons in such polymers during chemical doping processes can be monitored by electron spin resonance (ESR) spectroscopy [15,16]. 

In research on LC materials, conjugated polymers with an LC phase under appropriate conditions are of particular interest, offering both electrical conductivity and self-organized properties [17,18,19]. In the present study, a phenylene monomer bearing a pyrimidine-type LC group was synthesized, and the compound was demonstrated to exhibit both nematic and smectic phases. Copolymerization of this monomer with thiophene monomers was then conducted to afford π-conjugated LC copolymers. The optical properties, liquid crystallinity, and iodine doping effects of these π-conjugated LC copolymers were characterized as examples of a new type of aromatic LC conjugated polymer.

## 2. Experimental Section

### 2.1. Materials and Methods

4-[2-(4-Dodecyloxy-2-fluorophenyl)-pyrimidine-5-yl]-phenol (**1**, Midori Chemical Co.) was used for the LC side chain. ^1^H-NMR spectra were obtained with an EX-270 NMR spectrometer (JEOL). HMQC NMR was measured with AVANCE-600 NMR spectrometer (Bruker). ^19^F-NMR spectra were obtained with AVANCE-500 NMR spectrometer (Bruker). Molecular weights of the polymers were determined by gel permeation chromatography (GPC) with MIXED-D HPLC column (Polymer Laboratories), PU-980 HPLC pump (JASCO) and MD-915 multiwavelength detector (JASCO), with THF used as the solvent, with the instruments calibrated by polystyrene standard. Infrared spectroscopic measurements were carried out with FT/IR-300 spectrometer (JASCO). UV-vis absorption spectra and photoluminescence spectra of the polymers in chloroform were obtained with V-630 UV-vis optical absorption spectrometer (JASCO) and F-4500 fluorescence spectrophotometer (HITACHI). Concentration of the solutions was 0.1 mg/5 mL, and the excitation wavelength was set at absorption maximum wavelength (λ_max_) of π-π* transition of the main chain for each polymer. Optical texture observation was performed with ECLIPSE LV100 polarizing optical microscope (NIKON) equipped with TM-600PM (Linkam). Differential scanning calorimetry (DSC) analysis was carried out with DSC6200 (SEIKO Instruments). X-ray power was set at 800 mW in the XRD measurements. Free radicals of the polymer were detected with JES-TE200 ESR spectrometer (JEOL) during *in-situ* vapor phase doping process with iodine.

### 2.2. Monomer synthesis

**Scheme 1:**
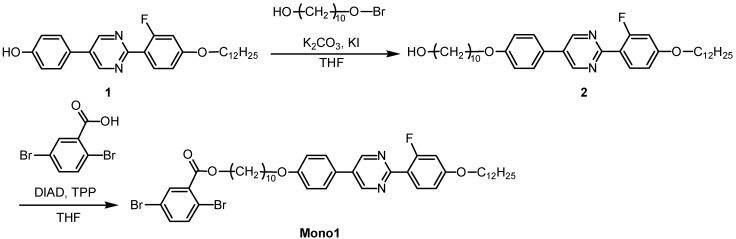
Synthesis of monomer bearing LC moiety.

10-[4-[2-(4-Dodecyloxy-2-fluorophenyl)-pyrimidine-5-yl]-phenoxy]-decan-1-ol (**2**) was prepared by Williamson etherification through a coupling reaction between **1** and 10-bromo-1-decanol (Scheme 1), as follows: compound **1** (0.925 g, 2.05 mmol), 10-bromo-1-decanol (0.40 g, 2.05 mmol), potassium carbonate (0.28 g, 2.05 mmol), and 18-crown-6-ether (0.05 g, 0.19 mmol) were mixed into 2-butanone (10 mL) and refluxed at 80 °C for 24 h to evaporate the solvent. The residue was then washed thoroughly with water and extracted with dichloromethane. Recrystallization from acetone followed by vacuum drying afforded the target product as a white solid (0.775 g, 1.28 mmol, yield = 62.3%). 

**Figure 1:**
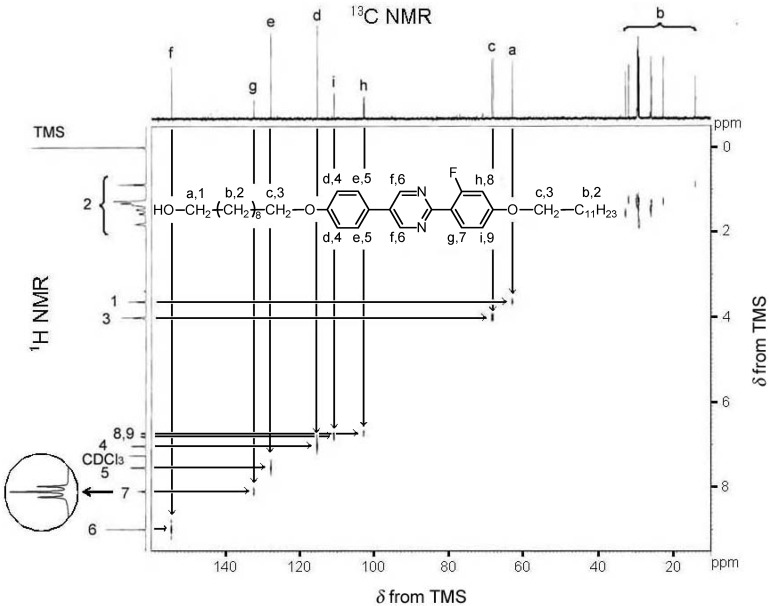
^1^H−^13^C hetero-nuclear multiple quantum coherence (HMQC) NMR of 10-[4-[2-(4-dodecyloxy-2-fluorophenyl)-pyrimidin-5-yl]-phenoxy]-decan-1-ol (compound **2**). The inset shows ^1^H-NMR signal of a proton positioned at 7.

The molecular structure of **2** was confirmed by ^1^H−^13^C hetero-nuclear multiple quantum coherence (HMQC) nuclear magnetic resonance (NMR) spectroscopy (Figure 1). 

Carbon atoms and protons adjacent to the hydroxy oxygen atom (a,1) exhibit correlated signals at 63.0 ppm (^13^C-NMR) and 3.65 ppm (^1^H-NMR), and signals due to carbon atoms and protons adjacent to the ether oxygen atom (c, 3) are observable at ca. 68.4 ppm (^13^C-NMR) and 4.00 ppm (^1^H-NMR), respectively. The carbon atom and proton adjacent to the fluorine atom (h, 8) exhibit double peaks due to spin coupling with the fluorine atom. Although proton no. 7 in Figure 1 (^1^H-NMR) apparently exhibits a triplet signal (inset), the signal may be a double doublet given that the proton is coupled with the adjacent proton and the remote fluorine atom with almost the same *J* value. ^1^H-NMR (600 MHz, CDCl_3_): *δ* 0.88 (t, 3H, *J* = 7.0 Hz), 1.27 (m, 26H), 1.47 (m, 4H), 1.57 (m, 2H), 1.81 (m, 4H), 3.64 (t, 2H, *J* = 6.3 Hz), 4.01 (t, 4H, *J* = 6.5 Hz), 6.73 (dd, 1H, *J* = 13.1, 2.3 Hz), 6.81 (dd, 1H, *J*_FH_ = 8.8 Hz, 2.3 Hz), 7.04 (d, 2H, *J* = 8.7 Hz), 7.55 (d, 2H, *J* = 8.7 Hz), 8.10 (t, 1H, *J*_HH_ = 8.8 Hz, *J*_FH_ = 8.8 Hz), 8.99 (s, 2H).

**Figure 2:**
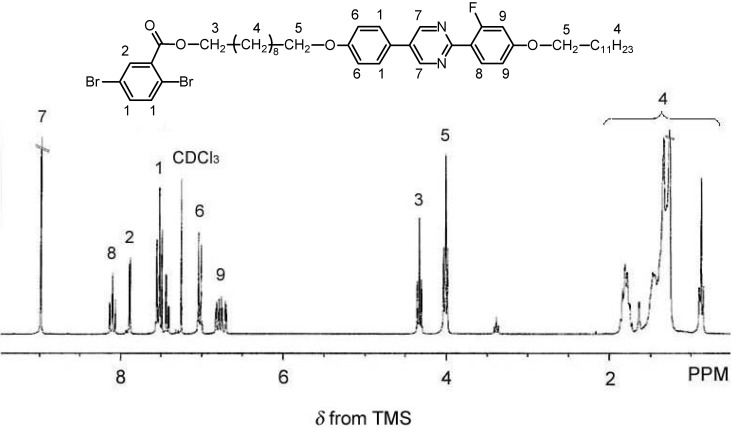
^1^H-NMR spectra of 2,5-dibromobenzoic acid 10-[4-[2-(4-dodecyloxy-2-fluorophenyl)-pyrimidin-5-yl]-phenoxy]-decyl ester (**Mono 1**).

The synthesis of 2,5-dibromo-benzoic acid 10-[4-[2-(4-dodecyloxy-2-fluorophenyl)-pyrimidine-5-yl]-phenoxy]-decyl ester (**Mono 1**) was carried out by a Mitsunobu coupling reaction between 2,5-dibromobenzoic acid and the hydroxy group (Scheme 1), as follows: 2,5-dibromobenzoic acid (0.24 g, 0.84 mmol), **2** (0.50 g, 0.82 mmol), and triphenylphosphine (0.25 g, 0.94 mmol) were mixed under nitrogen into tetrahydrofuran (THF; 7 mL), to which diisopropyl azodicarboxylate (DIAD, 40% in toluene; 0.46 g, 0.92 mmol) was added in a dropwise manner after cooling in an ice bath. The mixture was maintained at ca. 0 °C for 1 h, followed by stirring at room temperature for 10 h. After the reaction, the solvent was evaporated, and the residue was purified by column chromatography using silica gel and ethyl acetate. Recrystallization from acetone followed by vacuum drying afforded the target product as a white solid (0.55 g, 0.63 mmol, yield = 75.6%). Figure 2 shows the ^1^H-NMR spectra of **Mono 1**. 

The monomer exhibits signals corresponding to the LC moiety and dibromophenylene. ^1^H-NMR (270 MHz, CDCl_3_): *δ* 0.88 (t, 3H, *J* = 7.1 Hz), 1.30 (m, 32H), 1.81 (m, 4H), 4.01 (t, 4H, *J* = 6.5 Hz), 4.34 (t, 2H, *J* = 6.75 Hz), 6.77 (m, 2H), 7.03 (d, 2H, *J* = 8.9 Hz), 7.50 (m, 4H), 7.89 (d, 1H, *J* = 2.3 Hz), 8.10 (t, 1H, *J* = 8.9 Hz), 8.99 (s, 2H).

### 2.3. Polymerization

**Scheme 2:**
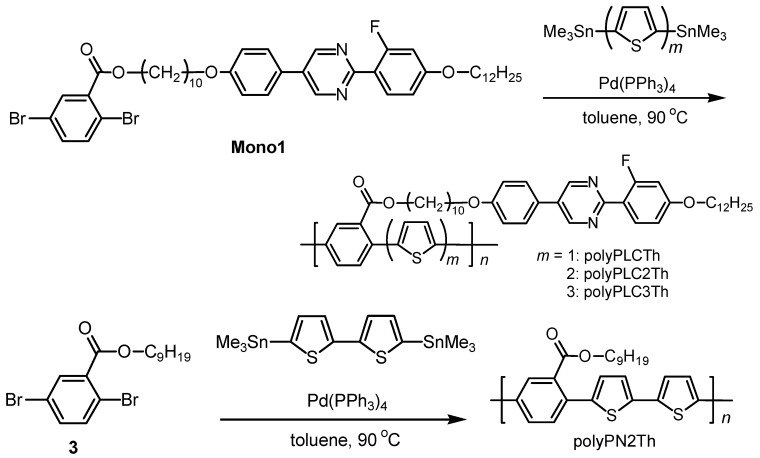
Synthesis of polymers.

Four different conjugated polymers were prepared via Stille polycondensation reactions of various thiophene monomers and **Mono 1** or 2,5-dibromobenzoic acid nonyl ester in the presence of a Pd(0) complex catalyst (Scheme 2). 

The four polymers are abbreviated polyPLCTh, polyPLC2Th, polyPLC3Th, and polyPN2Th, where P, LC, N, and Th denote the phenyl group, LC group, nonyl group, and thiophene, respectively, and the numbers denote the compositional number of thiophenes in the monomer unit.

The general procedure for synthesis of the polymers is as follows: **Mono 1** or 2,5-dibromobenzoic acid nonyl ester (0.058 mmol) and the thiophene monomer (0.058 mmol) were mixed into toluene (1 mL) under nitrogen and refluxed for 30 min. Tetrakis(triphenylphosphine)palladium(0) (Pd(PPh_3_)_4_) was then added to the mixture and the reaction allowed to proceed for 2 days at 90 °C. The reacted mixture was then dissolved in a minimal amount of chloroform and poured into a large amount of methanol under constant stirring. The solution was washed with methanol to remove the catalyst and the fractions of low molecular weight, and the solvent containing the undesired fractions was decanted. This procedure was repeated, and the remaining precipitate was collected by filtration and dried under vacuum to afford the desired product.

**Figure 3:**
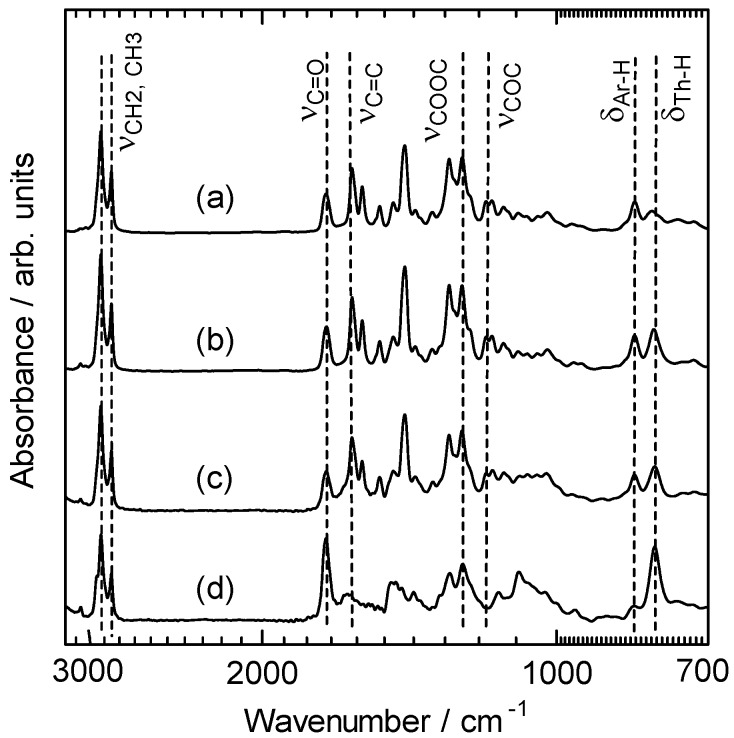
FT-IR spectra of the polymers. PolyPLCTh **(a)**, polyPLC2Th **(b)**, polyPLC3Th **(c)**, polyPN2Th **(d)**.

The polymerization results, including yield, molecular weight evaluated by gel permeation chromatography (GPC) relative to a polystyrene standard, and the ^19^F-NMR chemical shift from a trifluorotoluene standard, are summarized in Table 1. The polymers have a number-average molecular weight (*M*_W_) of 3,000 to 6,500. PolyPLCTh, polyPLC2Th, and polyPLC3Th exhibit ^19^F-NMR signals at 113 ppm, while polyPN2Th exhibits no signal in the ^19^F-NMR spectrum. This result confirms that the mesogenic group having the fluorine atom is introduced precisely into polyPLCTh, polyPLC2Th, and polyPLC3Th. Figure 3 shows Fourier transform infrared (FT-IR) absorption spectra for these four polymers. All of the polymers exhibit absorption bands assignable to the side chain attached to phenylene (e.g., –CH_3_ and –CH_2_– stretching at ca. 2,900 cm^−1^, C=O stretching at 1,720 cm^−1^, (CO)–O stretching at 1,250 cm^−1^, and C–O–C stretching at 1,170 cm^−1^). PolyPN2Th exhibits no absorption band related to the ether oxygen. The different constitution ratios of phenylene and thiophene moieties in the main chain of the polymers result in a variation in the intensity ratio of the signals due to Ar–H out-of-plane vibration at 835 cm^−1^ and Th–H out-of-plane vibration at 800 cm^−1^.

**Table 1:** Polymerization results.

Polymer	*M*_n_ (×10^3^)^a, b^	*M*_w_ (×10^3^)^a, c^	MWD^a^	Yield (%)^d^	^19^F *δ* (ppm)^e^
PolyPLCTh	4.9	6.5	1.3	94	113
PolyPLC2Th	3.6	4.5	1.3	89	113
PolyPLC3Th	4.2	5.6	1.3	81	113
PolyPN2Th	2.1	3.0	1.4	50	—

Polystyrene standard. Number average molecular weight. Weight average molecular weight. Calculated by (W_s_ / (W_p_ · W_m_) ) · 100, W_s_: weight of the polymer sample (g), W_p_: molecular weight of mru of the polymer (g/mol), W_m_: molar mass of the monomer (mol) *δ* from trifluorotoluene in ^19^F NMR.

## 3. Results and Discussion

### 3.1. Ultraviolet–visible absorption spectra

**Figure 4:**
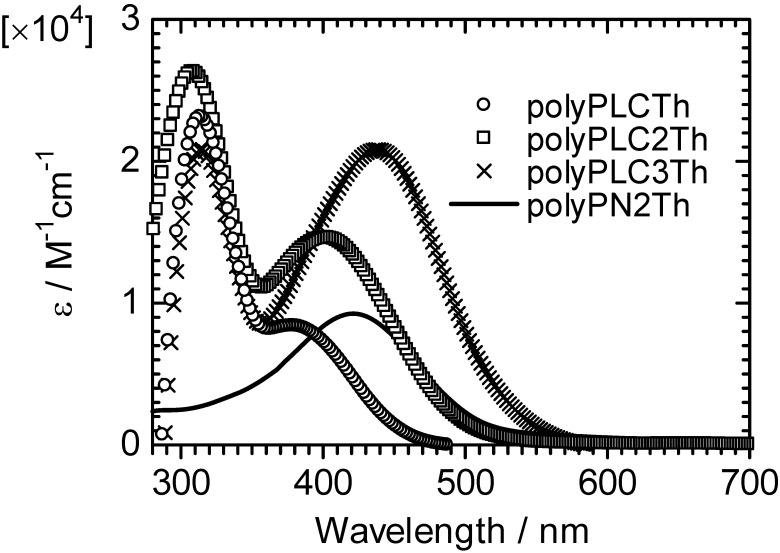
UV-vis optical absorption spectra of the polymers.

Figure 4 shows the ultraviolet–visible (UV–vis) optical absorption spectra of the polymers in chloroform solution. PolyPLCTh, polyPLC2Th, and polyPLC3Th exhibit absorption bands at ca. 310 nm, whereas no absorption band is observable at the same wavelength for polyPN2Th due to the absence of the mesogenic group as a side chain. This result suggests that the absorption band at 310 nm is assignable to the π–π* transition of the aromatic rings in the LC moiety. The polymers also display absorption bands at longer wavelengths. The absorption bands near 400 nm are attributable to the π–π* transition of the main chain (polyPLCTh, 379 nm; polyPLC2Th, 400 nm; polyPLC3Th, 438 nm; polyPN2Th, 421 nm), and the absorption intensities of the polymers increase with thiophene number in the monomer repeat unit of the polymers. These results indicate that increasing the thiophene number in the main chain results in extension of the effective conjugation length of the polymers.

### 3.2. Photoluminescence

**Figure 5:**
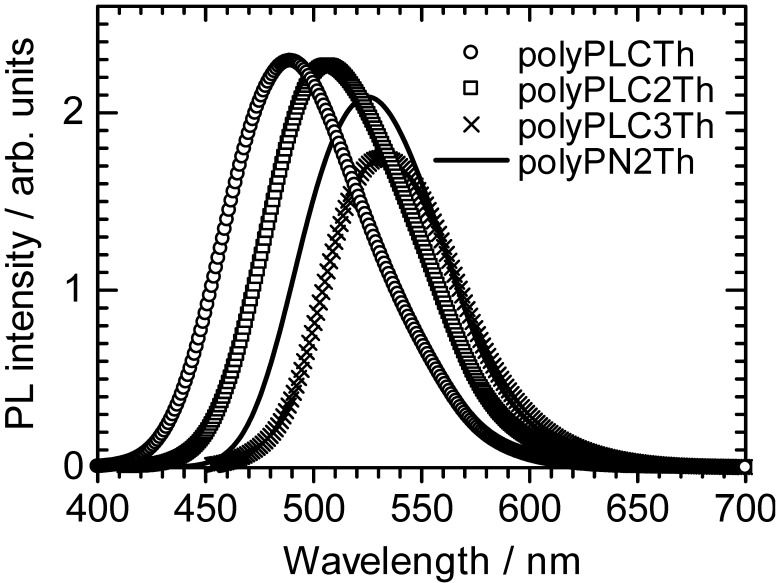
Photoluminescence spectra of the polymers.

Figure 5 shows the photoluminescence (PL) spectra for the four polymers in chloroform solution. The polymers exhibit PL maxima at 489 nm (polyPLCTh), 501 nm (polyPLC2Th), 532 nm (polyPLC3Th), and 524 nm (polyPN2Th). The shift in the PL position can be attributed to the extension of the effective conjugation length with increasing thiophene number in the monomer repeat unit, consistent with the UV–vis absorption results.

### 3.3. Polarizing optical microscopy observation

**Figure 6:**
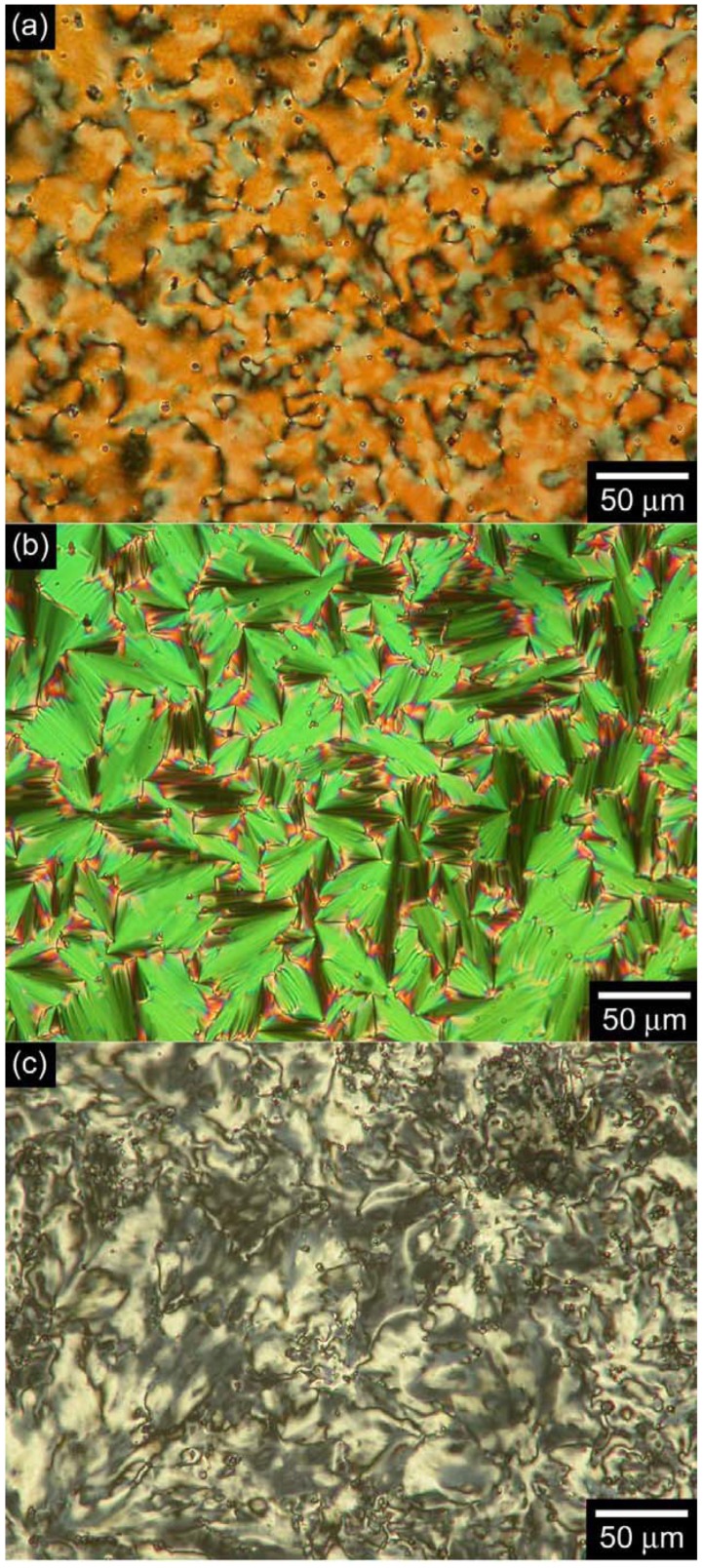
Polarizing optical microscopy (POM) images of LC phases of the monomer bearing LC moiety (**(a)**: 103 °C, **(b)**: 94 °C, **(c)**: 83 °C).

**Figure 7:**
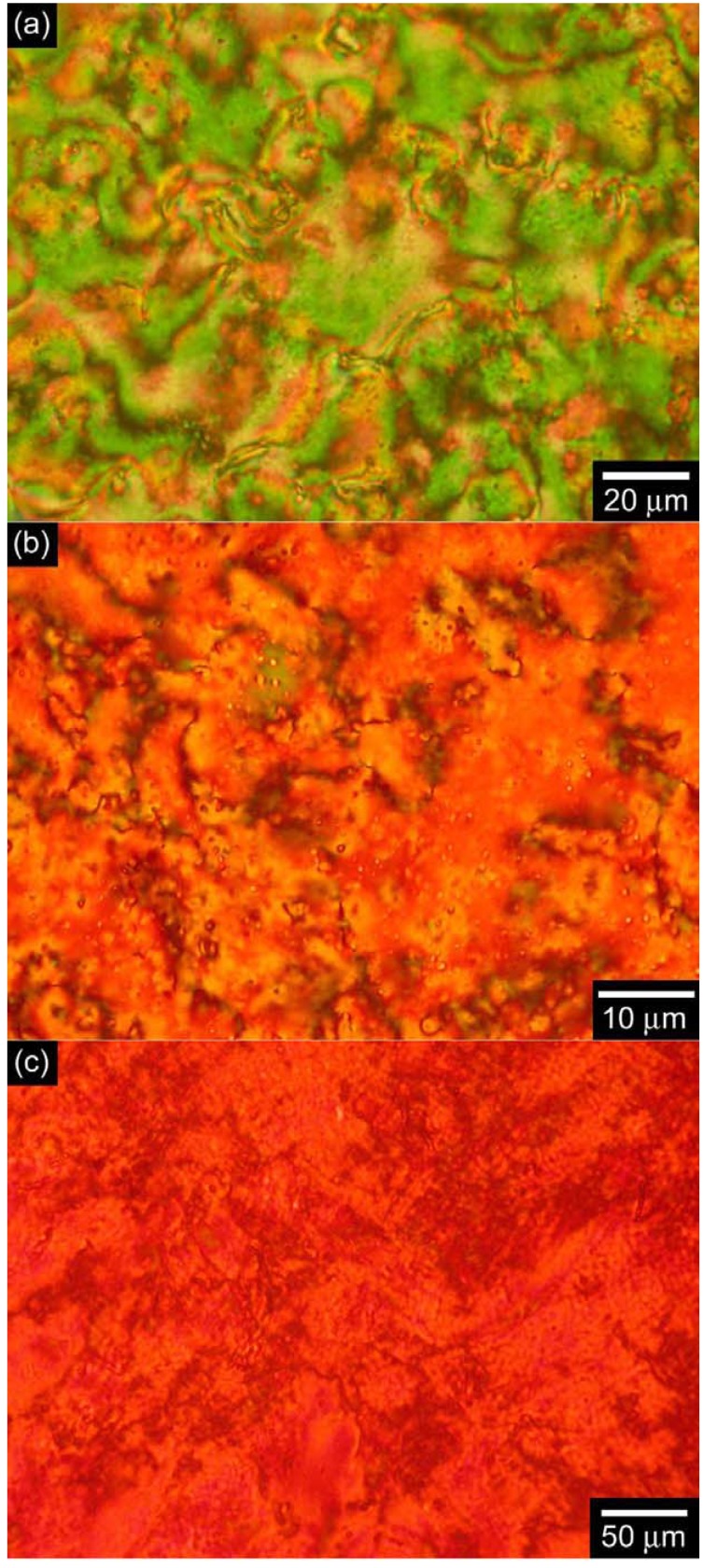
Polarizing optical microscopy (POM) images of LC phases of polyPLCTh (**(a)**: 156 °C) polyPLC2Th (**(b)**: 198 °C) and polyPLC3Th (**(c)**: 200 °C).

The optical textures of the monomer bearing the LC moiety and the corresponding polymers were observed by polarizing optical microscopy (POM). As shown in Figure 6, the monomer displays a Schlieren texture typical of the nematic (N) phase at 103 °C, a fan-shaped texture of the smectic A (SmA) phase at 94 °C, and a Schlieren texture of the smectic C (SmC) phase at 83 °C. Although the corresponding polymers do not display a smectic phase (Figure 7), the polymers exhibit Schlieren textures with higher LC-stable temperature range than that of the monomer. These results indicate that the polymers do not form the layered structure of the smectic phase, and that the substituents are oriented parallel to the main chain to afford a nematic LC phase. 

**Figure 8:**
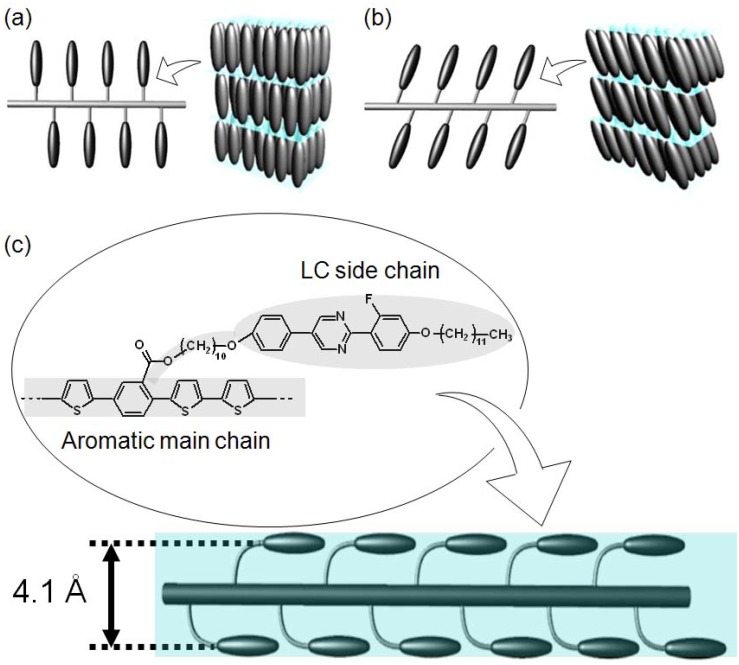
Possible structures of LC polymers: **(a)** SmA phase, **(b)** SmC phase, **(c)** N phase (inter-molecular distance (4.1 Å) was determined by x-ray diffraction).

The side chains are generally positioned perpendicular to the main chain in ordinary side-chain polymers [17], and the side chain molecules are able to tilt to form an SmC (tilt smectic) phase at appropriate temperatures (Figure 8). The mesogenic side chains of the polymers prepared in the present study, on the other hand, are oriented parallel to the main chain to form a nematic phase, and do not form a smectic LC phase. The side chains and main chain of the present polymer therefore appear to coordinate into a long cable-like mesogen as a main-chain/side-chain combination mesogen.

### 3.4. Differential scanning calorimetry

**Figure 9:**
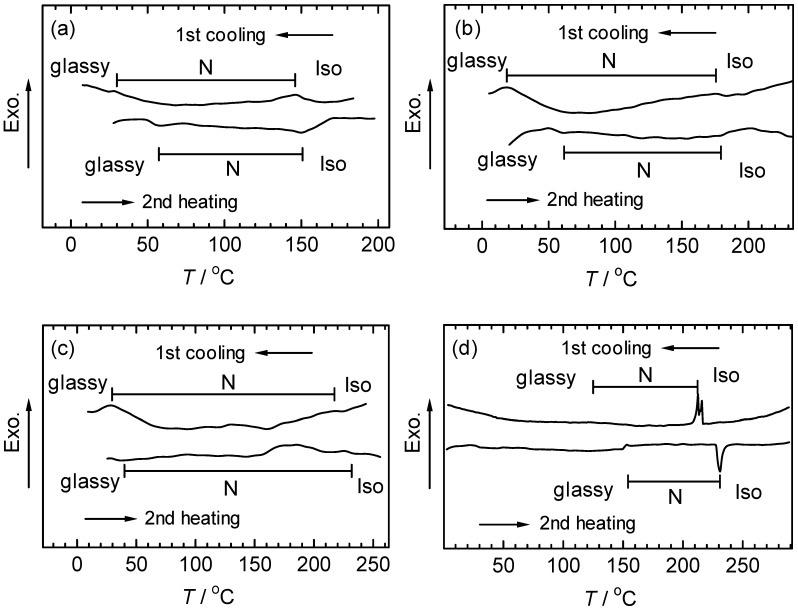
DSC curves of the polymers. PolyPLCTh (a), polyPLC2Th (b), polyPLC3Th (c), polyPN2Th (d).

Differential scanning calorimetry (DSC) of polyPLCTh, polyPLC2Th, and polyPLC3Th indicates broadenings of the phase transitions in the first cooling and second heating scans (Figure 9). The broadening of the phase transition points in the DSC curves can be attributed to the dispersity of molecular weights of the polymers, and also to individual phase transitions of the side chain and main chain. The phase transition temperatures between glassy state and the LC phase increase with compositional number of thiophene units in the monomer repeat units, suggesting that an increase in the rigidity of the main chain results in an increase in phase transition temperature. PolyPN2Th, on the other hand, exhibited sharp signals in the phase transition due to the absence of a mesogenic substituent, removing cooperation between the main chain and substituent in the development of the LC structure. In this case, the main chain functions only as a mesogen, and the polymer behaves as a typical main-chain polymer LC.

### 3.5. X-ray diffractometry

**Figure 10:**
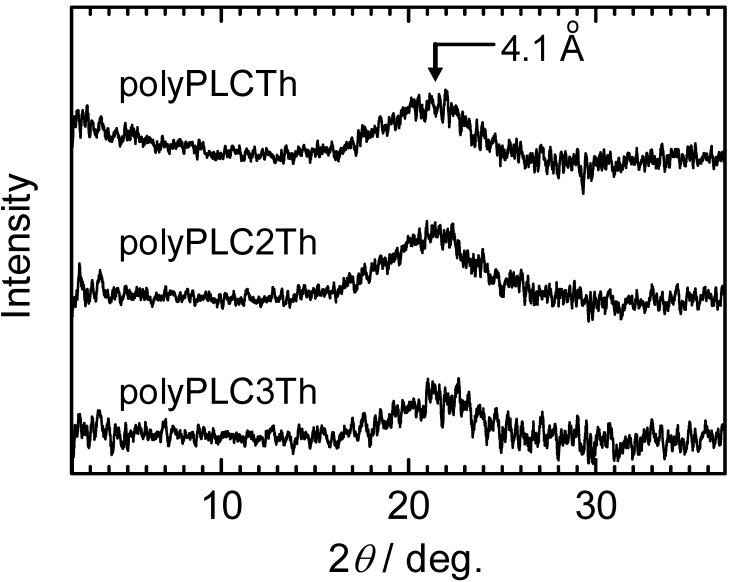
X-ray diffraction (XRD) pattern of the polymers at room temperature. The samples were obtained after cooling from the isotropic phase.

Figure 10 shows the x-ray diffraction (XRD) patterns for the three polymers. The polymer samples were heated once to produce the isotropic phase then cooled slowly to the glassy state in order to maintain the Schlieren texture of the polymers at room temperature prior to XRD measurements. The three polymers exhibit broad halos at the same angle (2*θ* = 21°, 4.1 Å), which is a typical pattern for nematic LC. Small-angle diffractions corresponding to the side chain length were not observed, confirming that these polymers do not have a layered structure, that is, the side chains are not located perpendicular to the main chain in these polymers. Although all of these polymers display the same XRD pattern, the distances between the adjacent LC side chains increase in the order polyPLCTh < polyPLC2Th < polyPLC3Th, indicating that the 4.1 Å distance corresponds to the intermolecular distance between the LC side chains parallel to the main chain. The side chain and main chain thus cooperate to form a single large mesogen as a supermolecular cable-like mesogen, consistent with the result that these polymers only adopt the nematic LC. The cooperation of side-chain and main-chain liquid crystallinity is therefore considered to be responsible for the formation of the nematic LC phase by these polymers, with no smectic LC formation despite the smectic LC phases exhibited by the monomer.

### 3.6. Electron spin resonance analysis

**Figure 11:**
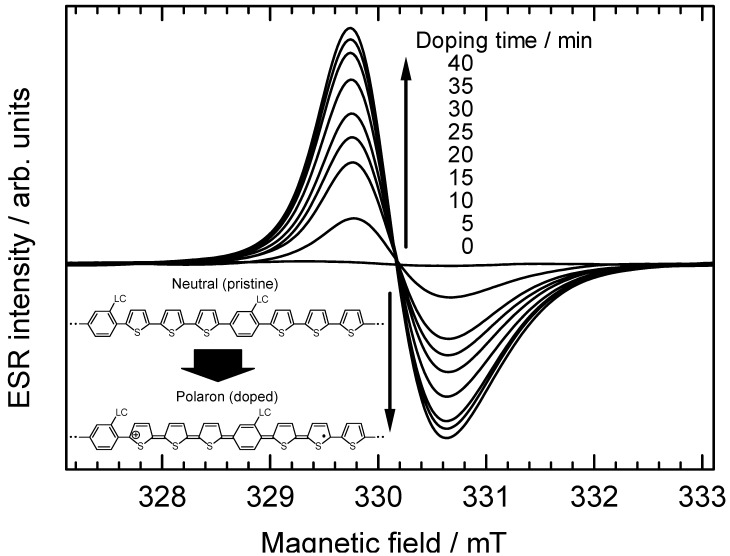
*In-situ* ESR spectra of polyPLC3Th during vapor phase doping of iodine.

**Figure 12:**
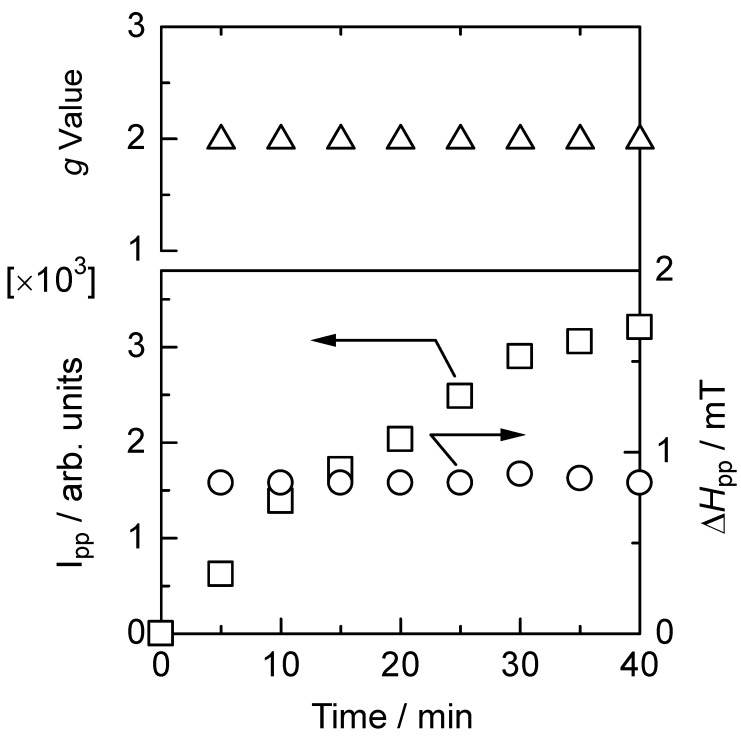
ESR intensity, *g* value, and linewidth of the ESR signal for polyPLC3Th during *in-situ* vapor-phase iodine doping.

ESR spectroscopy was employed to monitor free radical generation in polyPLC3Th during *in-situ* vapor doping of iodine (Figure 11). The pristine undoped polymer exhibits no ESR signal due to the absence of free radicals. Upon iodine doping, however, the intensity of the paramagnetic ESR signal increased with time, indicating that free radicals (conduction carriers) are generated by the doping process. The asymmetric lineshape of the ESR signal is consistent with the Dysonian pattern for organic conductive materials. The conduction carriers in the present case are radical cations (polarons). This result confirms the doping effect for the polymer, verifying that the polymers have a π-conjugated main chain. The *g* value, linewidth (Δ*H*_pp_), and ESR intensity of the polymer are plotted as functions of doping time in Figure 12. The *g* value and Δ*H*_pp_ remain unchanged during the doping process, whereas the ESR intensity increases with doping time, indicating that the density of conduction carriers increases with doping, while the carrier species remains unchanged. Heavy doping of the polymer would generate dications (bipolarons, charged carriers having no radicals) resulting in a decrease in ESR intensity.

## 4. Conclusions

A series of phenylene-thiophene-based π-conjugated copolymers bearing pyrimidine liquid crystal moieties were synthesized and confirmed to exhibit a nematic mesophase in the LC state. The liquid crystallinity of the polymers bearing the mesogen is produced by cooperation between the side-chain and main-chain liquid crystallinity. Vapor-phase doping of iodine was confirmed to generate radical cations on the polymer (polarons), with distinct Dysonian magnetic behavior typical of conductive polymers. The polymers prepared in the present study are potentially applicable as a new type of conductive LC material with self-alignment properties.

## Supplementary Materials

Supplementary File 1
