# Correction: Goto, H. *et al.* Liquid Crystalline π-Conjugated Copolymers Bearing a Pyrimidine Type Mesogenic Group. *Materials* 2009, *2*, 22-37

**DOI:** 10.3390/ma5010156

**Published:** 2012-01-09

**Authors:** Kohsuke Kawabata, Hiromasa Goto

**Affiliations:** Institute of Materials Science, Graduate School of Pure and Applied Sciences, University of Tsukuba, Tsukuba, Ibaraki, 305-8573, Japan

We found an error in the Scheme 1 in our paper published in *Materials* [1]. Corrected scheme is:

**Scheme 1:**
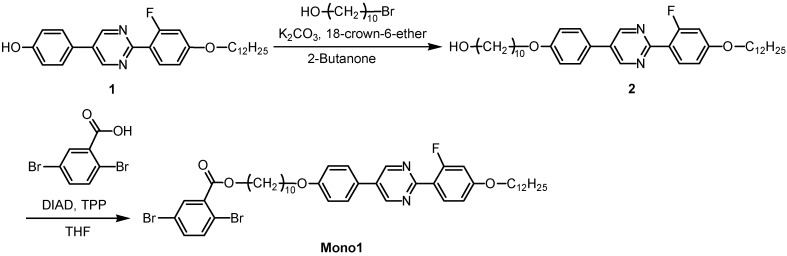
Synthesis of monomer bearing LC moiety.
