# Supplementary material for: Liquid Crystalline π-Conjugated Copolymers Bearing a Pyrimidine Type Mesogenic Group
Source: Materials (Basel). 2009 Jan 16;2(1):22–37. doi: 10.3390/ma2010022 (PMC5445693; doi:10.3390/ma2010022)
Supplement: Supplementary File 1 [file materials-02-00022-s001.pdf]

Materials **2012**, *5*, 156; doi:10.3390/ma5010156

OPEN ACCESS

materials

ISSN 1996-1944

www.mdpi.com/journal/materials

Correction

Correction: Goto, H. *et al.* Liquid Crystalline π-Conjugated Copolymers Bearing a Pyrimidine Type Mesogenic Group. *Materials* 2009, *2*, 22-37

Kohsuke Kawabata and Hiromasa Goto *

Institute of Materials Science, Graduate School of Pure and Applied Sciences, University of Tsukuba, Tsukuba, Ibaraki, 305-8573, Japan

* Author to whom correspondence should be addressed; E-Mail: gotoh@ims.tsukuba.ac.jp; Tel. +81-29-853-5128; Fax: +81-29-853-4490.

Received: 11 October 2011 / Accepted: 9 January 2012 / Published: 9 January 2012

We found an error in the Scheme 1 in our paper published in *Materials* [1]. Corrected scheme is:

Scheme 1. Synthesis of monomer bearing LC moiety.

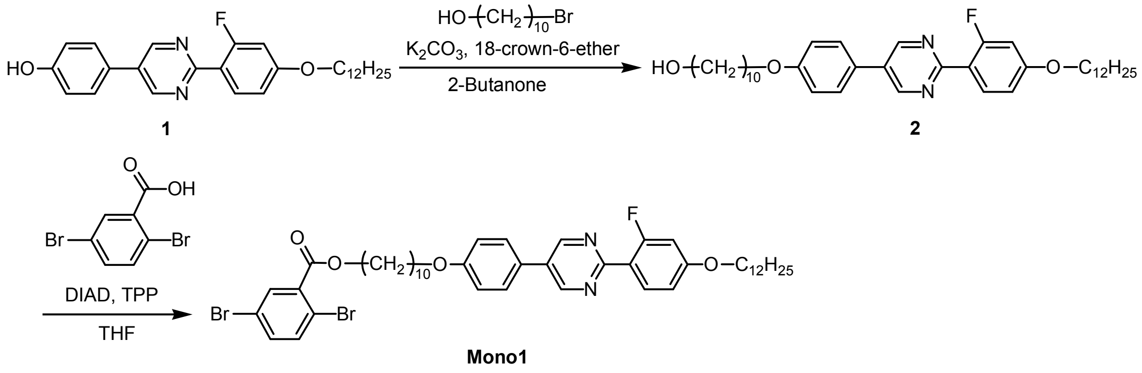

References

1. Kawabata, K.; Goto, H. Liquid Crystalline π-Conjugated Copolymers Bearing a Pyrimidine Type Mesogenic Group. *Materials* **2009**, *2*, 22-37.

© 2012 by the authors; licensee MDPI, Basel, Switzerland. This article is an open access article distributed under the terms and conditions of the Creative Commons Attribution license (http://creativecommons.org/licenses/by/3.0/).
